# Organ-on-a-Chip: Opportunities for Assessing the Toxicity of Particulate Matter

**DOI:** 10.3389/fbioe.2020.00519

**Published:** 2020-05-29

**Authors:** Jia-Wei Yang, Yu-Chih Shen, Ko-Chih Lin, Sheng-Jen Cheng, Shiue-Luen Chen, Chong-You Chen, Priyank V. Kumar, Shien-Fong Lin, Huai-En Lu, Guan-Yu Chen

**Affiliations:** ^1^Department of Electrical and Computer Engineering, College of Electrical and Computer Engineering National Chiao Tung University, Hsinchu, Taiwan; ^2^Institute of Biomedical Engineering, College of Electrical and Computer Engineering, National Chiao Tung University, Hsinchu, Taiwan; ^3^Ph.D. Degree Program of Biomedical Science and Engineering, National Chiao Tung University, Hsinchu, Taiwan; ^4^School of Chemical Engineering, University of New South Wales, Sydney, NSW, Australia; ^5^Bioresource Collection and Research Center, Food Industry Research and Development Institute, Hsinchu, Taiwan; ^6^Department of Biological Science and Technology, National Chiao Tung University, Hsinchu, Taiwan

**Keywords:** particulate matter, air pollution, respiratory health, cardiovascular effects, organ-on-a-chip

## Abstract

Recent developments in epidemiology have confirmed that airborne particulates are directly associated with respiratory pathology and mortality. Although clinical studies have yielded evidence of the effects of many types of fine particulates on human health, it still does not have a complete understanding of how physiological reactions are caused nor to the changes and damages associated with cellular and molecular mechanisms. Currently, most health assessment studies of particulate matter (PM) are conducted through cell culture or animal experiments. The results of such experiments often do not correlate with clinical findings or actual human reactions, and they also cause difficulty when investigating the causes of air pollution and associated human health hazards, the analysis of biomarkers, and the development of future pollution control strategies. Microfluidic-based cell culture technology has considerable potential to expand the capabilities of conventional cell culture by providing high-precision measurement, considerably increasing the potential for the parallelization of cellular assays, ensuring inexpensive automation, and improving the response of the overall cell culture in a more physiologically relevant context. This review paper focuses on integrating the important respiratory health problems caused by air pollution today, as well as the development and application of biomimetic organ-on-a-chip technology. This more precise experimental model is expected to accelerate studies elucidating the effect of PM on the human body and to reveal new opportunities for breakthroughs in disease research and drug development.

## Introduction

With the development of epidemiology in recent years, scientists have confirmed that airborne particulate matter (PM) is directly associated with respiratory pathology and mortality (Kim et al., 2018; Khaniabadi et al., 2019). For every 10 μg/m^3^ increase in PM_10_ concentration, respiratory system-related mortality increases by 0.58% (Analitis et al., 2006), and for every 10 μg/m^3^ increase in PM_2.5_, the incidence of respiratory-system related diseases increases by 2.07% (Zanobetti et al., 2009). These studies indicate that impaired lung function also increases the incidence and mortality of cardiopulmonary disease (Zanobetti et al., 2009; de Oliveira et al., 2012). In addition to the problem of increased risk of respiratory disease caused by compromised lung function, PM may also increase the incidence of lung cancer (Raaschou-Nielsen et al., 2016). In a large-scale study conducted in the United States with a sample of 188,699 non-smokers, each 10 μg/m^3^ increase in PM_2.5_ concentrations increased lung cancer-related mortality by 15–27% (Turner et al., 2011). Although PM is a global concern, severe air pollution episodes are often associated with industrialization, and urbanization. As China's rapid economic growth, several recent studies paid attentions to frequent air pollution episodes (Li et al., 2016; Lin et al., 2018). The air quality statistics report of Beijing from 2013 to 2015 shows that the 2 year average PM_2.5_ concentrations from 69 to 89 μg/m^3^ and the daily average concentrations ranged from 3 to 437 μg/m^3^ (Batterman et al., 2016). These studies have shown that it is imperative to address the harmful effects of PM on the human body, in addition to traditionally known respiratory diseases such as asthma and chronic obstructive pulmonary disease (COPD) (Hopke et al., 2019). The incidence of cardiopulmonary disease and lung cancer are also associated with a high mortality rate (Hamanaka and Mutlu, 2018). Therefore, it is essential to quickly and accurately elucidate the effects of PM on the human body, determine the causes of diseases, and formulate response strategies.

## Particulate Matter and Respiratory System

**Figure 1:**
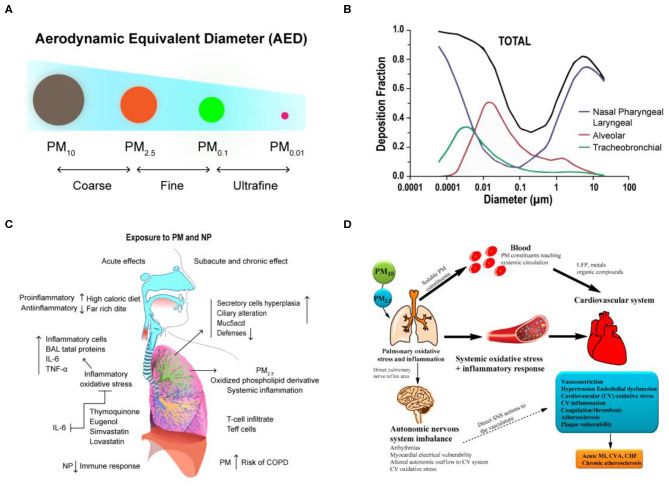
The transport and health effects of PM in human lung and cardiovascular. **(A)** Classification of PM size by aerodynamic equivalent diameter (AED). **(B)** PM deposition curves in the extrathoracic, bronchial, and alveolar regions for adult humans. Reproduced with permission from Tsuda et al. (2013). **(C)** The schematization of the immune responses and inflammation in lung cells by inhaled PM. Reproduced with permission from Nemmar et al. (2013). **(D)** There are three main hypotheses that PM can cause biological pathways for cardiovascular impairment. Reproduced with permission from Ngoc et al. (2018).

PM is one of the most important components of air pollution that affects human health and disease. It is classified based on the relative size, which is defined in terms of aerodynamic equivalent diameter (AED), not directly by the diameter of their actual particles. In other words, the size of the actual particles is converted into an equivalent diameter having the same aerodynamic properties (Raabe, 1976). PM can be divided into three AED levels based on the deposition and penetration ability of the particles in the human respiratory system: ≤ 10 μm, ≤ 2.5 μm, and ≤ 0.1 μm (PM_10_, PM_2.5_, and PM_0.1_, respectively) (Figure 1A). Particles with AED diameters >10 μm have a relatively small half-life in suspension and are mostly filtered by the nasal and upper respiratory tract, so researchers have classified PM >10 μm into three categories: (i) coarse PM (PM_2.5−10_), (ii) fine PM (PM_0.1−2.5_), and (iii) ultrafine PM (PM_0.1_) (Anderson et al., 2012).

Although the effects of PM exposure depend on individual physical characteristics, such as the pattern and rate of breathing, weight, and age, the size of the particles has been identified as a direct cause of health problems (Brown et al., 2013). Generally, the smaller the particles, the faster they penetrate and deposit in the deeper levels of the respiratory system. In nasal breathing, cilia and mucous membranes are very effective in filtering most particles larger than 10 μm in diameter. Because of the rapid deposition of PM with larger particle size, they tend to remain in the trachea or bronchi (upper respiratory tract). They initially accumulate in the nose and throat, and the body will eliminate these invasive PM through some reactive processes such as sneezing and coughing. Up to now, particles <10 μm in diameter are considered to have the greatest impact on human health, and because of their high penetration ability, they can evade the protective mechanisms of the upper respiratory tract and enter the alveoli deep in the lungs (Löndahl et al., 2006; Kim et al., 2015). Computer simulations have shown that particles with diameters between 1 and 10 μm are primarily deposited in the nasopharyngeal and laryngeal of upper airway regions, and particles with diameters between 1 and 100 nm are deposited in the lower bronchial and alveolar region, where gas exchange occurs (Tsuda et al., 2013) (Figure 1B). However, particles smaller than 1 μm tend to behave like gas molecules, so it is extremely easy for them to penetrate into the alveoli and affect gas exchange in the lungs and even penetrate the barrier of the lungs and enter the circulatory system, further migrating to other cells, tissues, or circulatory and metabolic systems and leading to serious health problems (Table 1).

**Table 1:** Effects of PM on the human respiratory system.

**Diameter (μm)**	**Distribution characteristics**	**Effects on human physiology**
PM_10_	Deposits in nose and throat	Can cause allergic rhinitis, cough, asthma, and other symptoms
PM_2.5−10_	Deposits in upper nasal cavity and deep respiratory tract	Causes fibrous paralysis, bronchial mucus hypersecretion, and mucosal gland hyperplasia leading to reversible bronchospasm, inhibits deep breathing and spreading to bronchi
PM_2.5_	Less than 10% deposits in bronchi, ~20–30% deposits in lungs	Can cause chronic bronchitis, bronchiole expansion, pulmonary edema, bronchial fibrosis, or other symptoms
PM_0.1_	Deposits inside alveolar tissue	Promotes significant increase in macrophages in the lungs, causes emphysema and alveolar destruction

Currently, there have been many studies on the biological mechanism and effects of PM on the respiratory system (Xing et al., 2016), which primarily involve the following aspects: (1) Functional injury caused by free radical peroxidation. Studies have shown that free radicals, metals, and organic components in PM_2.5_ can induce the free radical formation, cause oxidation of lung cells, and also cause the production of reactive oxygen species (ROS), resulting in DNA damage and cell death (Donaldson et al., 1996; Lodovici and Bigagli, 2011). (2) Imbalance of intracellular calcium regulation (calcium homeostasis). Calcium ion is a physiological index of cell function regulation. However, free radical and ROS reactions induced by PM_2.5_ can cause abnormal intracellular calcium concentrations, which lead to apoptosis and necrosis (Li et al., 2015; Al Hanai et al., 2019). (3) Inflammatory injury. This is also the most extensive part of current studies. PM_2.5_ can cause significant immune responses and inflammation in lung cells (Nadeau et al., 2010; He et al., 2017). These inflammatory responses to Th1 and Th2 triggered by Toll-like receptor (TLRs) pathways can lead to activation of neutrophils, T cells, and alveolar macrophages, which is also associated with asthma and COPD (Nemmar et al., 2013) (Figure 1C). Although studies on PM and its biological mechanisms are currently ongoing, an important future research direction is to elucidate the inflammatory response to PM in the lungs.

## Particulate Matter and Cardiovascular Effects

With the development of epidemiology in recent years, scientists have validated the effects of long-term PM exposure on the respiratory system. PM can penetrate deep into the trachea and bronchi and can even deposit in alveolar tissue. Hydroxyl radicals (·OH) produced from reactive oxygen species (ROS) through activated metals are the main factors that cause oxidative damage to DNA. If damaged DNA is not repaired in a timely matter, it can induce cancer or lead to other irreversible damage (Valavanidis et al., 2005). Environmental exposure to this fine PM not only causes respiratory disease but also affects heart rate, blood pressure, vascular tone, blood coagulation, and formation of atherosclerotic lesions (Suwa et al., 2002; Bennett et al., 2018; Huang et al., 2018). In addition, there is a positive correlation between fine PM and the incidence and mortality of cardiopulmonary disease. The World Health Organization (WHO) reported that 3.7 million deaths in 2012 were due to air pollution, which accounted for 6.7% of deaths worldwide. Of these, 16% were deaths due to lung cancer, 11% were due to chronic obstructive pulmonary disease and associated diseases, 29% were due to heart disease and stroke, and about 13% were due to respiratory infections. In addition, fine PM in the air that is inhaled into the lungs can translocate to the bloodstream and be transported to the blood vessels and the heart, which can induce arrhythmia and reduce myocardial contractility and coronary blood flow (Nemmar et al., 2003). The possible mechanisms for cardiopulmonary risk following inhalation of fine PM into the lungs can be roughly classified into three groups: (1) Stimulating the production of inflammatory factors: inducing the secretion of inflammatory factors such as cytokines, activated immune cells, platelets, and endothelin, from basal cells in the lungs (Kido et al., 2011; Tsai et al., 2012). (2) Translocation of PM: toxic effects caused by translocation of PM or its components to the circulatory system (Nemmar et al., 2002). (3) Neuroendocrine disorders: the balance of the autonomic nervous system or heart rate is affected by the binding of PM to receptors located on the lungs or nerves (Ngoc et al., 2018; Snow et al., 2018) (Figure 1D).

Du *et al*. summarized recent research results on short- and long-term exposure to PM_2.5_ (Du et al., 2016). The results show that every 10 μg/m^3^ increase in short-term PM_2.5_ exposure concentration increases overall mortality and cardiovascular-related mortality by 0.4–1.0%, while every 10 μg/m^3^ increase in long-term exposure increases overall mortality by 10% and cardiovascular-related mortality by 3–76%. With respect to various cardiovascular diseases, PM_2.5_ has the greatest impact on coronary heart disease, moderate impact on heart failure and stroke, and the smallest impact on peripheral vascular diseases and arrhythmia. Because these risk factors not only cause a sharp increase in the risk of cardiovascular disease, and the metabolic syndrome secondary to it is closely associated with some of the most significant causes of death worldwide, including increased risk of Alzheimer's disease, Parkinson disease, dementia, and stroke (Fu et al., 2019). Although the true pathogenic mechanism is currently unknown and the impairment of cardiopulmonary function due to fine PM is due to a complex series of effects, the establishment of an *in vitro* model that represents the human body in a large number of studies is an urgent need. It is expected that establishing an *in vitro* model of cardiopulmonary function will yield new possibilities and opportunities for understanding the hazards and influencing mechanisms associated with environmental engineering and human health.

## Assessment for Biological Toxicity of Fine Particulate Matter

**Figure 2:**
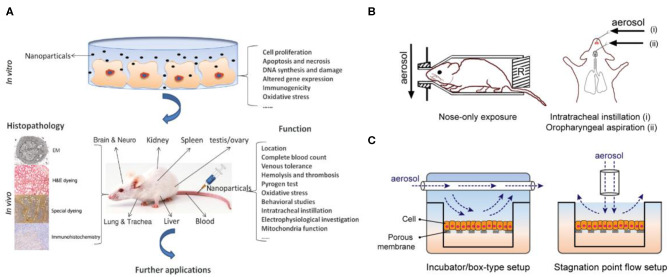
Two main methods for elucidating the mechanisms of PM toxicity. **(A)** *In vitro* and *in vivo* toxicity assessment of PM. Reproduced with permission from Yang et al. (2017). **(B)** *In vitro* animal experiments exposed to aerosols mainly include nose exposure, intratracheal instillation, and oropharyngeal aspiration. Reproduced with permission from Fröhlich and Salar-Behzadi (2014). **(C)** Air–liquid interface (ALI) *in vitro* models for investigating respiratory research.

Epidemiological and clinical studies have linked exposure to fine PM to adverse health outcomes, which may also be associated with increased mortality and morbidity in various cardiopulmonary diseases. Despite much evidence of the effects of PM on human health, the causes of physiological responses and the changes and damage to cellular and molecular mechanisms have not yet been fully explained. There are currently two main methods for elucidating the mechanisms of PM toxicity (Fröhlich and Salar-Behzadi, 2014; Yang et al., 2017) (Figure 2A). One is the use of *in vivo* animal models to evaluate the effects of fine PM on the respiratory and cardiovascular systems (Figure 2B). The other type is *in vitro* cell experiment models (Figure 2C); the use of various *in vitro* models has proven valuable for studying the molecular and cellular mechanisms behind different physiological effects more deeply.

Studies using animal models have demonstrated the effects of fine PM exposure on different organs and the incidence of different diseases. With respect to acute reactions, most studies have focused on inflammatory responses, and relatively few researchers have investigated specific responses to disease (Hong et al., 2016; Wang H. et al., 2017). Conversely, with respect to chronic reactions, a large number of disease-related findings have been reported, including DNA damage, lung parenchyma damage, pulmonary fibrosis, and granuloma formation (Xu et al., 2013; Shadie et al., 2014). Meanwhile, because *in vitro* models have demonstrated that cell experiments are the most suitable model for investigating mechanisms of toxicity, such as initiation events for inflammatory effects or genotoxicity, these data cannot be explained because of genetic differences in animal experiments (Wang J. et al., 2017). In view of this, the genotoxicity and biological indicators linking disease and cancer have been clearly recognized in particular, but this is still a challenge in other applications, which also includes further elucidation of the mechanism of fine PM toxicity in humans (Vawda et al., 2013).

Although current knowledge does not fully understand the health effects of PM exposure, studies over the past decade have suggested the potential cytotoxicity. It has been observed in cell studies that PM stimulation has caused cell viability decline, cell death, ultrastructural disruptions, genetic toxicity (mutagenicity and DNA damage), and oxidative stress (Peixoto et al., 2017). These mechanisms involved in the inflammatory response, including the up-regulation of cytokines downstream of the caspase cascade and the kinase pathway, the up-regulation of metal-redox sensitive transcription factors NF-κβ and AP-1 (Øvrevik et al., 2015). In addition, the PM also increases inflammatory mediator-related gene expression and protein secretion, such as TNF-α, IL-1β, IL-8, IL-6, and MCP-1 (Fuentes-Mattei et al., 2010; Ryu et al., 2019). On the other hand, similar reactions have been observed in animal experiments, showing DNA damage, oxidative stress and inflammatory mediator-related protein secretion and recruitment of inflammatory cells in many organs (de Oliveira et al., 2018). In addition, many studies have shown association between exposure to PM and chronic diseases. These adverse health effects include asthma, chronic obstructive pulmonary disease (COPD), atherosclerosis, diabetes and allergic sensitization (Guo et al., 2018; Li et al., 2018).

Mammalian cell culture studies are often used as the first step in toxicity evaluation, cell-based studies are still greatly limited with respect to the complex structures of physiological mechanisms in humans, and it is impossible to simulate the complex conditions and the interrelated physiological information of the entire organism. Animal experiments play an important role in PM research, where they allow *in vivo* toxicological testing by exposing animals to various PM environments via the oral and dermal routes. Although animals can inhale PM and develop comprehensive systemic outcomes, there is often a large difference between mechanistic and genetic indicator data and clinical outcomes. This is primarily due to differences among species and their physiological functions, such as differences in respiratory rate between experimental mice and humans (Curbani et al., 2019), as well as the problems of genetics, low throughput, high cost, and ethical concerns.

As yet, there is currently no ideal experimental model for study the toxicity of fine particulate matter since both *in vitro* and *in vivo* models have limitations. Notably, the interpretation of chronic toxicity studies is relatively lacking of information, which requires consideration of whether the information obtained from animal studies is similar to human responses. This issue may be expected to be overcome through the advancement of biotechnology and biomedical engineering technologies, thereby obtaining a useful *in vitro* model that allow long-term cultivation of functional responses that express the human organ. In addition, due to the diversity and regional differences of PM composition, how to systematically study the toxicology of PM on the human body (including single components and the interaction between components) is also the focus of future research (Jia et al., 2017; Park et al., 2018).

## Organs-ON-Chips

With respect to the effects of PM exposure on the human body, many studies have demonstrated the relationship between PM and health risk, but further understanding of the mechanisms of human toxicity is still lacking. The problems faced are derived from the major differences in genes and structures between the species used in animal experiments and humans. Although biomimetic technology experiments are the most appropriate model for investigating mechanisms of toxicity, only a complete description and definition of genotoxicity and indicators between disease and cancer have been made at present, and other aspects are still being researched. In this field, improving the representativeness of *in vitro* experiments and strengthening the reference value of data has become important issues in research. This topic has been discussed by other authors (Vanderburgh et al., 2017; Ahadian et al., 2018; Costa and Ahluwalia, 2019). Developments and progress in biomimetic technology will bring unlimited potential for breaking through the research bottlenecks faced in this field.

**Figure 3:**
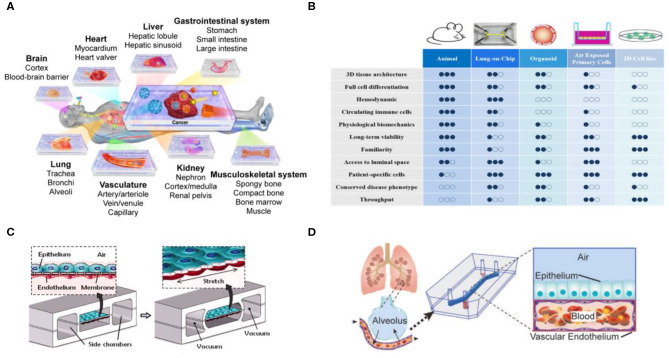
Organs-on-chips technology for tissue model development**. (A)** Organs-on-chips platform provides an *in vitro* model of various organs. Reproduced with permission from Zhang et al. (2017). **(B)** Comparison of experimental strategies for current *in vitro, in vivo*, and Organs-on-chips models. Adapted with permission from Nawroth et al. (2019). **(C)** A lung-on-a-chip microdevice reproduce human physiological respiratory movements. Reproduced with permission from Huh et al. (2010). **(D)** Construction of a lung-on-a-chip with tissue/organ-level physical microstructure and microenvironment. Reproduced with permission from Jain et al. (2018).

To resolve the great differences between animal experiments and clinical trials, techniques have been developed in recent years for construction of organs-on-chips, with the goal of replacing animal experiments and achieving more accurate and reliable preclinical data (Alépée et al., 2014; Zhang et al., 2017, 2018) (Figure 3A). Currently, organ-on-a-chip development mostly relies on materials with high biocompatibility for construction of a 3D microenvironment suitable for cell growth so that cells can establish cell-to-cell interactions that are not possible in most 2D cell culture environments, to observe the phenomena of simulated organs more accurately. In particular, this new organ-on-a-chip models provide unlimited potential to replicate critical tissue-tissue responses by reconstructing dynamic physiological forces, cellular microenvironments, and 3D structures of human organ. The development of organ-on-chips and in-depth descriptions have been heatedly discussed in other reviews (Rothbauer et al., 2018; Nawroth et al., 2019) (Figure 3B). In addition, when combined with the “induced pluripotent stem cell (iPSCs)” technique in somatic cells, it is possible to successfully differentiate individualized target tissues of interest without traumatizing the organs. Researchers have successfully integrated various iPSC-derived cells with organ-on-chips, such as blood vessels-on-chip, a blood–brain barrier-on-chip and heart-on-chip systems. The different methods for creating organ-on-chips using stem cells also have been described in depth by other groups (Geraili et al., 2018; Jodat et al., 2018; Cochrane et al., 2019).

## Lung-On-A-Chip

Although different organs-on-chips have their own requirements, applications associated with the respiratory tract are always very strong. For example, when the lungs are infected by fine PM, bacteria, or viruses, white blood cells accumulate, and the mucus produced block the airway. These processes are difficult to observe in animals and further highlight the importance of developing lung-on-a-chip technology. The Wyss Institute at Harvard University has been a worldwide pioneer in the development of *in vitro* organ-on-a-chip, and the lung-on-a-chip they developed was the first in the world (Huh et al., 2010) (Figures 3C,D). It is entirely based on polydimethylsiloxane (PDMS) material, with an upper and a lower layer of channels separated by a porous membrane coated with extracellular matrix. In its internal structure, the upper layer consists of alveolar epithelial cells that allow gases to pass through, and the lower layer consists of microvascular epithelial cells that allow white blood cells to pass through, thus simulating lung function. In 2016, Benam et al. used this technology to test smoking and non-smoking conditions, and **c**onfirmed that using the lung-on-a-chip yielded experimental results that were closer to clinical physiological and inflammatory reactions compared with those from animal experiments, and previously undiscovered biomarkers that were even more accurate were found and analyzed (Benam et al., 2016a). At the same time, other teams have developed lung chip models with different design structures and physiological responses (Fishler et al., 2015; Fishler and Sznitman, 2016; Humayun et al., 2018; Stucki et al., 2018; Khalid et al., 2020). The lung-on-a-chip have been developed to demonstrate their importance in drug development and disease models, but still have several practical challenges must be overcome if such devices are to be used in toxicology research and application (Low and Tagle, 2017; Wu et al., 2020). The aim of overcoming these challenges is to improve the usability of these devices and to simulate metabolism in the human body more accurately.

## Heart-On-A-Chip

In addition, evidence from animal studies has shown that nanoparticles can cross the alveolar-capillary barrier and subsequently deposit in extrapulmonary organs such as the vasculature and heart (Choi et al., 2010). Using specific organ chips, such as heart-on-a-chip to investigate the toxicity of PM may also have great potential value. The heart-on-a-chip is mainly used to study electrical stimulation (Xiao et al., 2014), cardiac electrophysiology (Sidorov et al., 2017) and disease models (Wang et al., 2014). Marsano et al. recently established heart-on-a-chip platform integrates mechanical stress and 3D matrix microenvironment, showing better differentiation and electromechanical coupling of the iPSC-derived cardiomyocyte (Marsano et al., 2016). Liu *et al*. demonstrated the latest bioelectronic heart-on-a-chip model, which can regulate the concentration of oxygen through the microfluidic channel, and integrated bioelectronic devices to successfully monitor the cardiac electrophysiology responses to acute hypoxia (Liu et al., 2020). These examples fully demonstrate that heart-on-a-chip may provide greater ability to recapitulation the cellular microenvironment and tissue function. In the future, it is expected that suitable heart-on-a-chip models can be selected to investigate the human toxicity investigation of PM deposition, penetration and metabolism, but it is necessary to consider whether to choose particles with corresponding size, composition and complex for stimulation or exposure assessment. Of particular interest are the mechanisms of PM-mediated toxicity on the systemic health effects. In recent years, with the gradual advancement of multi-organ chips technology, it is expected to provide more clues to accelerate the clarification of the human toxicity of PM deposition, penetration and metabolism (Yuancheng et al., 2018; Carvalho et al., 2019), especially for the specific examples of lung- heart- on chip model, which could be used to investigate the systemic toxicology of PM into the human body.

## Opportunities and Challenges

It is worth mentioning that the organ-on-a-chip not only shows the application value in research, but also the establishment of related companies has started to appear in the past 5 years (Mastrangeli et al., 2019), such as TissUse GmbH, Emulate, Inc., MIMETAS Inc., Nortis, Inc., AlveoliX AG., Hesperos Inc. In addition, the U.S. Food and Drug Administration (FDA) announced in April 2017 that it had signed a multi-year cooperation agreement with Emulate Inc. (spinoff from the Wyss Institute for Biologically Inspired Engineering at Harvard University), and will begin a series of trials using organ-on-a-chip technology to develop a testing platform for toxicological safety assessment (Isoherranen et al., 2019). These results demonstrate the potential of applying organ-on-a-chip systems to human health assessments. In the future, the organ-on-a-chip technology is able to integrate stem cell technology, microenvironment and personalization parameters (e.g., breathing pattern, heart rate, substance abuse, etc.) to allow the construction of models of different genders, regions, ages, and diseases to minute minor physiological differences, thereby promoting the development of precision health (van den Berg et al., 2019).

Despite the progress made with organ-on-a-chip models, there remains a question that the organ-level functional replication is limited by the source of cells. In the case of pulmonary alveolar model, the aspect of long-term culture of primary human alveolar type I and type II epithelial cells is particularly challenging limitation (Shiraishi et al., 2019; Weiner et al., 2019). Therefore, the organ-on-a-chip technology faces limited availability and the inability to expand primary cells, requiring the establishment of cell cultures directly from donors and patients, which will increase the cost of experiments and the difficulty of popularizing the technology. On the other hand, the most organ-on-a-chip are made of PDMS due to their high biocompatibility, oxygen permeability, and transparency. The PDMS chip devices can directly match conventional cell culture incubators and biological microscopes. However, a large amount of protein molecules will be adsorbed on the surface of PDMS (Wong and Ho, 2009; Gokaltun et al., 2017), which results in that the supplement or stimulating substance of the cell culture cannot fully interact with the cells. To avoid adsorption of non-specific proteins, some teams have used polylactic acid (PLA) (Ongaro et al., 2020), poly (methyl methacrylate) (PMMA) (Nguyen et al., 2019), polystyrene (PS) (Lee et al., 2018), and polycarbonate (PC) (Henry et al., 2017), and more advantages and limitations of PDMS materials also have been introduced in detail by other teams (Halldorsson et al., 2015; Gokaltun et al., 2017).

## Multi-Organ Chips

**Figure 4:**
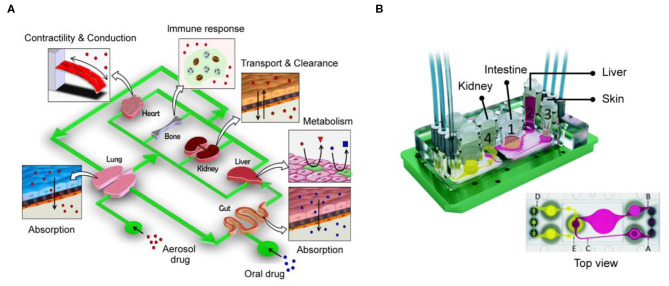
Integrate multi-organ chip platforms to create complex interactions between human organs. **(A)** The design concept of the human body chip. One of the most promising *in vitro* system for replicating the systemic responses of human body. Reproduced with permission from Huh et al. (2011). **(B)** Four-organs-on-a-chip system employed intestine, liver, skin, and kidney tissue that proportionately simulated the physiological environment of the human body. Reproduced with permission from Maschmeyer et al. (2015).

With the development of organs-on-chips in the past decade, the establishment of single types of organ-on-a-chip or the development of disease models on a chip has gradually matured. Although the organ-on-chips have seen great progress, it is not enough to rely on a simulation single organ model for a comprehensive understanding due to the highly complex interactions between human organs. In 2004, Dr. Shuler and colleagues first proposed the concept of reproducing human physiological functions in chip devices (Sin et al., 2004). The increasing demand for *in vitro* models, chips integrating multiple organs have become a major topic in recent years, and they also represent a major step forward in organ-on-a-chip technology (Figure 4A). Currently, chips capable of representing multiple organs in an integrated manner and fully and accurately simulating human tissue are still being developed (Skardal et al., 2017; Oleaga et al., 2018; Boos et al., 2019; Sung et al., 2019; Zhao et al., 2019). For example, a new model for physiological pharmacokinetics (PKs) and pharmacodynamics (PDs) has successfully predicted the clinical patient data of cisplatin PDs. This model is linked through fluidically coupled vascularized organ chips to investigate PK and PD parameters of oral and injectable drugs (Herland et al., 2020). It is worth noting that the experiments of this model have reached an automated system through robotic fluidic coupling of multiple organ chips, and maintained the long-term culture of organ-specific functions for 3 weeks (Novak et al., 2020). The automated multi-organ chip system integrated with high-throughput screening has the potential to improve the prediction of drugs (or other foreign substances) absorption, distribution, metabolism, excretion and toxicity for clinical trials.

As another example, the device in a recent study mainly integrates four tissues—liver, heart, muscle, and neurons (Oleaga et al., 2016). It is composed of the liver (which serves to process drug metabolites and drug processes drugs or prodrugs), heart (which is the most important organ in the human body), skeletal muscle (which is responsible for glucose storage levels in the body), and neurons (which represent a particularly sensitive cell system). After culturing this system in a continuous flow environment for 14 days, its feasibility and functionality were demonstrated, and because the cells used in the system were primary cells and cells derived from iPSCs, they exhibited the exchange of metabolites and signaling molecules. In addition, by measuring heart rate, muscle contractility, neuroelectrophysiology, and production of liver albumin and urea, it served as an accurate model for predicting toxicity in multiple human organs. In another study, Maschmeyer et al. integrated pre-formed bowel and skin models into a hepatic spheroid and renal epithelial barrier tissue model, establishing a microchannel system that could support the functions of four types of organs in a co-culture over a long period of 1 month (Maschmeyer et al., 2015) (Figure 4B). In addition, this four-organs-on-a-chip system employed a structure that more proportionately simulated the physiological fluid and tissue environment of the human body. It simulated drug absorption and metabolism in the small intestine, metabolism by the liver, and excretion by the kidneys, which are all key factors that determine the efficacy and safety of drug treatments. These systems allow us to further understand metabolic and genetic analyses and provide an alternative to systemic toxicity testing. In addition, these examples demonstrate that integrated multi-organ chips are an important part in the ability to simulate complex reactions and interactions between tissues, whether in drug testing, toxicological screening, or construction of organ-on-a-chip models.

Therefore, integrating multi-organ chips are expected to replace the inadequacies of traditional *in vitro* models, promoting studies of the effects of air pollution on the body and the early development of drugs, as these devices are designed to mimic the physiological structure of internal organs and interactions with soluble metabolites, thereby achieving *in vitro* the interactive effects between organs. However, current multi-organ chip models are mainly used for systemic processes of oral and injectable drugs, but lacks models for PM inhalation. In a recent human inhalation study, Miller et al. investigated the transport behavior of gold nanoparticle inhaled into the lung (Miller et al., 2017). The results showed that the blood and urine of the volunteers still found gold nanoparticle after 3 months of exposure, indicating systemic retention and delayed urinary excretion. This study clearly understands the ability of inhaled nanoparticles to penetrate lung tissue, but investigating the interactions between human organs, especially for the cardiopulmonary system remains a challenge. Based on the most direct impact of PM on cardiopulmonary function, in the future, it is urgent to form an integrated platform by connecting the organ chips of the lung and heart in the future. Even PM gas can be exposed to such a platform for discussion. It is hoped that the cardiopulmonary function model established *in vitro* can be used to obtain new possibilities and opportunities for PM analysis, so that it can more effectively clarify the impact of PM on the human body *in vitro* and find out the causes of cardiopulmonary diseases.

## Outlook

In addition to well-known respiratory diseases such as COPD, fine PM in the air that is inhaled into the lungs are translocated to the bloodstream and transported to the blood vessels and heart, where they induce arrhythmia, reduce myocardial contractility, and reduce coronary blood flow, thereby increasing the incidence and mortality of cardiopulmonary diseases. Related studies have shown that the harmful effects of fine PM on health may reach an uncontrollable point by 2030. Therefore, it is essential to quickly and accurately elucidate their effects on the human body, determine the causes of disease, and formulate response strategies.

Although epidemiological and clinical studies have produced much evidence of the effects of fine PM on human health, it has not yet been fully explained how physiological responses and cellular and molecular mechanisms of change and injury are caused. Currently, most health evaluation studies of fine PM are conducted through cell culture or animal experiments. Cell-based studies are still greatly limited compared to the complex structures of physiological mechanisms in humans, and it is impossible to simulate the complex conditions and the interrelated physiological information of the entire organism. Animal experiments play an important role in studies on fine PM, where they allow *in vivo* toxicological testing by exposing animals to various fine PM environments via the oral and dermal routes. Although animals can inhale fine PM and develop comprehensive systemic outcomes, there is often a large difference between mechanistic and genetic indicator data and clinical outcomes. This is primarily due to differences among species and their physiological functions, such as differences in respiratory rate between experimental mice and humans, as well as the problems of genetics, low throughput, high cost, and ethical concerns. These reasons have caused difficulty when investigating the causes of air pollution and associated human health hazards, the analysis of biomarkers, and the development of future pollution control strategies. Organ-on-a-chip biomimetic technology will bring unlimited potential for breaking through the bottlenecks faced in previous studies.

Reviewing the current development of organ-on-chips, most research focuses on drug development and disease models (Huh et al., 2012; Esch et al., 2015; Benam et al., 2016b). Except for the toxicological applications of lung-on-a-chip and cigarette smoking, other integrated studies related to environmental PM have not been extensive. According to previous reviews (see the section on Particulate matter and respiratory system and Particulate matter and cardiovascular effects), there are several clues worthy of attention such as DNA damage, inflammatory injury and PM translocation. For these research topics, it is believed that there is a great opportunity to obtain more undiscovered information by applying current organ-on-chips and multi-organ chips technology. For example, DNA damage and inflammatory injury could refer to related research on drug toxicity testing, translocation of PM could refer to related research on nanoparticle drug delivery, and further research on chronic toxicology could refer to the multi-organ chips model with PKs and PDs parameters. On the other hand, organ-on-a-chip systems have been shown to be closer to clinical physiology and inflammatory response, compared to traditional experimental model approaches. It has the potential to be a useful *in vitro* model for investigating the relationship between PM and related diseases. Therefore, the successful development of *in vitro* chips for simulating organs is a necessary avenue toward modern assessment of the health effects of air pollution. Its rapid and efficient screening capabilities are expected to help governmental agencies and the clinical sector move toward the correct policy and drug development routes, reduce costs, and significantly shorten the process of drug and foreign substance toxicity testing (Ronaldson-Bouchard and Vunjak-Novakovic, 2018).

**Figure 5:**
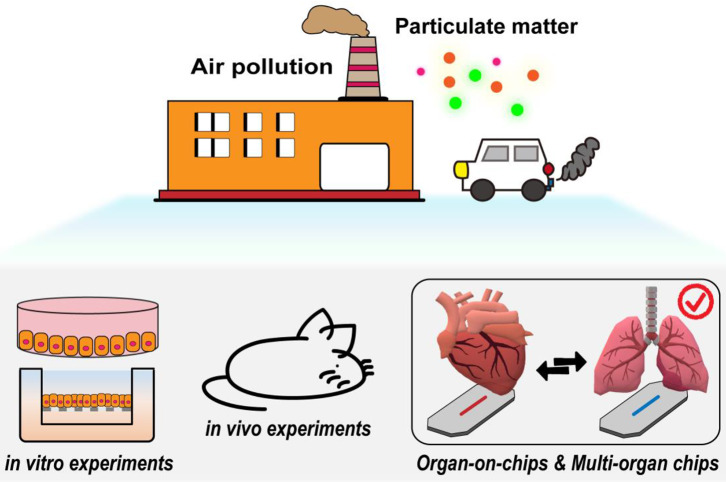
The potential value of organ-on-a-chip biomimetic technology for PM toxicity. Its 3D microenvironment and biomimetic circulating air/liquid dynamic environment are expected to be used for PM health assessment.

The technology is still evolving from single organ to multi-organ chips, it is expected to be realized as a long-term and highly active cardiopulmonary chip. Its 3D microenvironment and more biomimetic cyclic dynamic environment combined with fine PM are expected to be applied to health evaluation, physiological indicators, creation of cardiopulmonary disease models, and drug testing. This more precise experimental model is expected to replace existing cell culture or animal experiments and accelerate studies elucidating the effect of fine PM on the human body (Figure 5).

## Author Contributions

All authors contributed toward conceptualization, preparation, and validation of the manuscript.

## Conflict of Interest

The authors declare that the research was conducted in the absence of any commercial or financial relationships that could be construed as a potential conflict of interest.
